# The Role of Cardiolipin in Cardiovascular Health

**DOI:** 10.1155/2015/891707

**Published:** 2015-08-02

**Authors:** Zheni Shen, Cunqi Ye, Keanna McCain, Miriam L. Greenberg

**Affiliations:** ^1^Department of Biological Sciences, Wayne State University, Detroit, MI 48202, USA; ^2^Department of Biochemistry, The University of Texas Southwestern Medical Center, 5323 Harry Hines Boulevard, Dallas, TX 75390-9038, USA

## Abstract

Cardiolipin (CL), the signature phospholipid of mitochondrial membranes, is crucial for both mitochondrial function and cellular processes outside of the mitochondria. The importance of CL in cardiovascular health is underscored by the life-threatening genetic disorder Barth syndrome (BTHS), which manifests clinically as cardiomyopathy, skeletal myopathy, neutropenia, and growth retardation. BTHS is caused by mutations in the gene encoding tafazzin, the transacylase that carries out the second CL remodeling step. In addition to BTHS, CL is linked to other cardiovascular diseases (CVDs), including cardiomyopathy, atherosclerosis, myocardial ischemia-reperfusion injury, heart failure, and Tangier disease. The link between CL and CVD may possibly be explained by the physiological roles of CL in pathways that are cardioprotective, including mitochondrial bioenergetics, autophagy/mitophagy, and mitogen activated protein kinase (MAPK) pathways. In this review, we focus on the role of CL in the pathogenesis of CVD as well as the molecular mechanisms that may link CL functions to cardiovascular health.

## 1. Introduction

Cardiolipin (CL) is the signature lipid of mitochondrial membranes. It contains two phosphatidyl moieties joined by a central glycerol backbone, forming a dimeric structure [1]. Thus, unlike other phospholipids that contain two fatty acyl chains linked by glycerol, CL has four acyl chains. Considering the potential number of combinations of fatty acyl groups, a very large number of CL species may be possible. Interestingly, in most organisms and tissues, the fatty acyl composition of CL is unique and specific. In humans, CL acyl species vary in different tissues, but the most abundant species in the heart is tetralinoleoyl-CL [2]. While CL plays critical roles in mitochondrial biogenesis, fusion and fission, respiration, and protein import [3], it is also involved in various cellular processes outside of the mitochondria. These include, but are not limited to, cell wall biogenesis [4], vacuole homeostasis [5], ageing [6], the cell cycle [7], and apoptosis [8]. In this review, we focus on the role of CL in the pathogenesis of CVD as well as the molecular mechanisms that may link CL functions to cardiovascular health.

## 2. CL Synthesis

**Figure 1:**
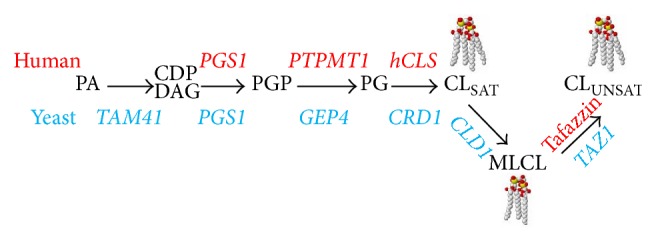
Cardiolipin synthesis and remodeling pathway in humans and yeast. Phosphatidic acid (PA) is converted to CDP-diacylglycerol (CDP-DAG) by CDP-DAG synthase. Phosphatidylglycerolphosphate synthase catalyzes the conversion of CDP-DAG to phosphatidylglycerolphosphate (PGP), which is dephosphorylated to phosphatidylglycerol (PG). PG is converted to unremodeled CL with mostly saturated acyl chains (CL_SAT_). CL_SAT_ is deacylated to monolyso-CL (MLCL) by phospholipases and MLCL is reacylated to CL with mostly unsaturated acyl chains (CL_UNSAT_). The genes encoding human enzymes are indicated in red, and genes that encode yeast enzymes are in blue.

Unlike mitochondrial membrane lipids that are synthesized in the endoplasmic reticulum,* de novo* synthesis of CL occurs exclusively in the inner membrane of the mitochondria [9], in a series of well-characterized steps that are highly conserved from yeast to higher eukaryotes [10]. As shown in Figure 1, the first step in the CL biosynthetic pathway is the conversion of phosphatidic acid (PA) to CDP-diacylglycerol (CDP-DAG), which is catalyzed in the inner membrane by CDP-DAG synthase encoded by* TAM41* [11–13] in yeast.* PGS1* encoded phosphatidylglycerolphosphate synthase catalyzes transfer of the phosphatidyl group from CDP-DAG to a glycerol-3-phosphate molecule to generate phosphatidylglycerolphosphate (PGP) [14, 15]. PGP is subsequently dephosphorylated to phosphatidylglycerol (PG) by PGP phosphatase [16, 17], encoded by* PTPMT1* in mammals [18, 19] and* GEP4* in yeast [20]. The final step in the biosynthetic pathway is carried out by CL synthase, encoded by* hCLS1* in human cells [21–23] and by* CRD1* in yeast [24–26]. In this step, a second phosphatidyl group is added to PG from another CDP-DAG molecule, generating unremodeled CL [9, 23, 27].

The acyl composition of CL varies in different tissues, due primarily to CL remodeling following* de novo* synthesis. CL remodeling may occur through two mechanisms (Figure 1) [28]. In the two-step mechanism, CL is first deacylated to monolyso-CL (MLCL) by phospholipases [29]. In yeast, the only CL-specific phospholipase is encoded by* CLD1* [30] while in mammals, several phospholipases are reported to have CL-hydrolyzing activities, including iPLA_2_ *β*, iPLA_2_ *γ*, cPLA_2_, and sPLA_2_ [31–33]. MLCL is then reacylated to remodeled CL by the transacylase tafazzin, encoded by the tafazzin gene (*TAZ/G4.5*) located on Xq28 in human cells [34] and by* TAZ1* in yeast [35, 36]. Acyltransferases encoded by* ALCAT1* [37] and* MLCLAT1* [38] have also been described in mammalian cells. In the one-step mechanism, CL remodeling occurs by direct transacylation [39, 40]. Mutations in tafazzin perturb CL remodeling and cause the life-threatening genetic disorder Barth syndrome (BTHS) [41], which is discussed below.

## 3. Relationship between CL and CVD

### 3.1. Cardiomyopathy

#### 3.1.1. Barth Syndrome

The most direct link between CVD and CL is seen in Barth syndrome (BTHS), an X-linked genetic disorder of CL remodeling caused by tafazzin mutations. BTHS manifests clinically as cardiomyopathy, skeletal myopathy, neutropenia, and growth retardation [42]. Biochemical phenotypes include decreased levels of CL, increased MLCL, and altered CL fatty acyl composition [43–45]. More than 160 mutations in the tafazzin gene have been identified in BTHS patients [46–48]. Interestingly, there is a wide disparity of clinical phenotypes, even among patients with the same mutation, ranging from being asymptomatic to death of newborns. Thus, some patients with an increased MLCL/CL ratio appear asymptomatic [49]. A study in which mutated BTHS tafazzin proteins were expressed in the yeast* taz1Δ* mutant reported that 18 of 21 BTHS proteins did not restore MLCL levels to normal, as expected [50]. However, expression of 3 of the 21 BTHS proteins restored MLCL levels in the yeast* taz1Δ* mutant to normal. In typical cases, total CL is decreased to about 80% in BTHS platelets and skeletal muscle and 20% in cardiac tissue [44]. CL species vary in different tissues. Tetralinoleoyl-CL (L4-CL) is the most abundant CL species in heart, skeletal muscle, and most other tissues, whereas acyl species such as arachidonic and docosahexaenoic acids are found in brain [51, 52]. L4-CL is absent in BTHS, while increases in other CL species are found [43–45]. As mentioned, tafazzin deficiency results in decreased CL, increased MLCL, and altered CL species, any of which may cause the pathology in BTHS. Recent findings in yeast indicate that deletion of Cld1-mediated deacylation rescues growth and lifespan defects in tafazzin-deficient cells [53, 54]. Because the* CLD1* mutation restored CL levels without generating remodeled CL, these findings suggest that, at least in yeast, decreased total CL and/or increased MLCL but not decreased remodeled CL leads to defects associated with tafazzin deficiency. If this is true in BTHS cells, inhibiting CL deacylation may, thus, be a novel potential strategy to treat BTHS patients.

#### 3.1.2. Diabetic Cardiomyopathy

Diabetes is a metabolic disease characterized by increased levels of glucose in the blood over a prolonged period. It is due to poor insulin production (type I) or insulin resistance with *β*-cell dysfunction (type II) [55]. Diabetic complications include a group of diseases derived from microvascular and macrovascular damage, including diabetic cardiomyopathy, myonecrosis, stroke, peripheral vascular disease, nephropathy, retinopathy and encephalopathy [56]. Diabetes doubles the risk of CVD, of which diabetic cardiomyopathy is the leading cause of mortality. Diabetic cardiomyopathy is characterized by altered lipid composition and mitochondrial dysfunction in the diabetic myocardium [57]. In the early stages of pathological development in the type II diabetic mouse model, a sharp decrease in total cardiac CL is observed [58]. In addition to a decrease in the whole cell CL content, there is also a shift from the predominant fatty acyl species, L4-CL (18 : 2), to longer and polyunsaturated fatty acids, due to aberrant CL remodeling [58, 59]. Strikingly, these alterations are similar to changes observed in the type I model of diabetes. In type II diabetic mice treated with the antidiabetic drug rosiglitazone, the wild type CL profile in the heart was restored, as total CL and L4-CL increased, and polyunsaturated CL decreased [60]. Impairment of CL synthesis plays a causal role in mitochondrial dysfunction [61–63], and mitochondrial dysfunction is associated with the pathogenesis of diabetic CVD, especially with the sequential events following silent myocardial ischemia in diabetics [64]. Thus, the sharp decrease in total cardiac CL and the altered CL fatty acyl species in the early stages of diabetic pathogenesis may play a key role in the progression of this disease.

### 3.2. Myocardial Ischemia-Reperfusion Injury

Myocardial ischemia occurs when the myocardium does not receive sufficient blood flow, resulting in irreversible injury and cell death [65]. Restoration of circulation in the ischemic myocardium leads to reperfusion injury [65]. Ischemia-reperfusion injury causes diverse myocardial dysfunctions, including cardiac contractile abnormalities [66–68], abnormal left-ventricular pressure [69], arrhythmia [70–72], and increased occurrence of ventricular fibrillation [73, 74].

In the early stages of myocardial ischemia, there is an increase in reactive oxygen species (ROS). During and after ischemia-reperfusion, ROS is thought to trigger lipid peroxidation as well as damage to cellular macromolecules and the electron transport chain which, together, lead to apoptosis, necrosis, and tissue damage [75–77]. Unsaturated CL acyl species in the mitochondrial inner membrane that are close to the site of ROS generation are vulnerable to oxidative damage. Consistent with this, total CL was decreased and peroxidized CL was increased in the rat heart during ischemia-reperfusion [78]. A study of ischemia-reperfusion in rabbit heart reported that reduction of total CL was due in large part to a significant decrease in CL in the subsarcolemmal mitochondria, whereas CL in the interfibrillar mitochondria was unchanged [79]. The levels of all other phospholipids remained unaffected. Decreased CL was proposed to be the cause of decreased enzyme activities of electron transport chain complexes I [80], III [81], and IV [78] in the rat heart ischemia-reperfusion model. The enzyme activities were restored by the addition of exogenous CL, but not by other phospholipids or peroxidized CL [78]. In summary, a feedback loop appears to be formed, in which CL is damaged by ischemia-reperfusion-induced ROS, and damaged CL leads to impairment of electron transport chain complexes, resulting in the generation of more ROS. CL also directly binds to cytochrome c (Cytc), and CL-bound Cytc has peroxidase activity that can produce CL hydroperoxides [82]. A known factor that stimulates the activity of this CL/Cytc peroxidase is increased H_2_O_2_ [83]. Peroxidized CL has a much lower affinity for Cytc [84]. In addition, several studies show that apoptosis factors, t-Bid and Bax, preferentially localize to the inner and outer membrane contact sites, which are rich in CL [85–87]. t-Bid binding and Bax insertion at the contact sites cause irreversible membrane permeabilization and promotes release of Cytc into the cytosol [87, 88], resulting in apoptosis.

### 3.3. Atherosclerosis

Atherosclerosis is a form of arteriosclerosis in which an artery wall thickens due to chronic invasion and further accumulation of white blood cells, remnants of dead cells, cholesterol, and triglycerides [89]. Oxidized CL (oxCL) is found to accumulate both in rabbit and human atherosclerotic lesions [90] and in the aortic root of mice fed a high fat diet [91]. Increased anti-oxCL IgG [92–94] and IgM [93, 95] antibodies are associated with atherosclerosis development. oxCL is recognized as a natural antigen that stimulates proinflammatory effects in the artery and promotes formation of atherosclerotic plaques [92, 96]. However, some studies purport that autoantibodies to oxCL may serve a protective role against the onset and development of atherosclerosis [97, 98]. The discrepancies regarding the effects of anti-oxCL antibodies on atherosclerosis may reflect the influence of potential physiological modifiers, including age, gender, and other existing diseases. The anticoagulation protein annexin A5 has been reported to bind to and inhibit the proinflammatory effects of oxCL [99], providing the basis for a potential therapeutic strategy for oxCL positive atherosclerosis.

### 3.4. Emerging Relationships between CL and Dilated Cardiomyopathy with Ataxia Syndrome (DCMA), Heart Failure (HF), and Tangier Disease

#### 3.4.1. DCMA

Dilated cardiomyopathy with ataxia (DCMA) syndrome is an autosomal recessive genetic disorder that is characterized by early onset dilated cardiomyopathy with conduction defects, nonprogressive cerebellar ataxia, testicular dysgenesis, growth failure, and 3-methylglutaconic aciduria [100]. The clinical manifestations of DCMA are similar to those found in BTHS. Patients with DCMA have a common mutation, a G→C base substitution within a splice site of the* DNAJC19* gene [100]. DNAJC19 protein localizes to the mitochondria and shares sequence and location similarity with yeast Tim14, an essential subunit of the TIM23 complex [101, 102]. TIM23 is required for the import of protein precursors from the cytoplasm into the mitochondrial matrix and inner membrane [103]. This suggests that the DCMA phenotype may result from defective mitochondrial protein import. As the loss of CL also leads to defective mitochondrial protein import [104–108], it is interesting to speculate that defective import of specific mitochondrial proteins may be common to DCMA and BTHS.

A recent study suggests that CL may play a role in the pathogenesis of DCMA [109]. DNAJC19 protein is reported to form a PHB/DNAJC19 complex with prohibitin, a ring-like scaffold protein located in the mitochondrial inner membrane. The PHB/DNAJC19 complex modulates CL remodeling by regulating tafazzin activity. siRNA-mediated knockdown of* DNAJC19* did not affect CL or MLCL levels but altered the acyl chain composition of CL [109], while knockout of* PHB2* resulted in reduced total CL, accumulated MLCL, and altered CL species. These data suggest that the PHB/DNAJC19 complex plays a role in CL synthesis and remodeling. However, whether the cause of DCMA is due to defective protein import, altered CL fatty acyl species that results from loss of* DNAJC19*, or a combination of the two remains unknown.

#### 3.4.2. HF

Heart failure (HF) results from inability of the heart to pump blood with normal efficiency, resulting in edema, shortness of breath, and lack of energy. HF is usually the end stage of CVD, including cardiomyopathy, heart attack, cardiac valvular disease, atrial fibrillation, and high blood pressure [110]. In both the spontaneously hypertensive HF rat model (SHHF) and human HF patients, decreased tafazzin mRNA levels were observed, concomitant with compensatory increases in the activity of phosphatidylglycerolphosphate synthase and MLCL acyltransferase [111]. However, studies of the CL profile in HF are controversial. While most studies report a significant reduction of total CL and L4-CL in human HF [112–114] and in the rat HF model [112, 115], one study reported an unchanged CL profile in a rat model with intracoronary microembolization-induced HF [116]. It is likely that different HF pathogenesis mechanisms lead to varying degrees of CL profile change and mitochondrial damage.

#### 3.4.3. Tangier Disease

Tangier disease (TD) is a genetic disorder of cholesterol efflux and lipid metabolism characterized by a nearly complete absence of plasma high-density lipoproteins (HDLs), atherosclerosis, peripheral neuropathy, and an increased risk for developing CVD [117, 118]. The genetic cause of TD is the mutation of the ABCA1 gene, which is located on chromosome 9 [119]. ABCA1 encodes a highly conserved ATP-binding cassette transporter. The ABCA subfamily of ABC transporters is involved in lipoprotein metabolism and lipid transport across the plasma membrane [120]. Researchers propose that a physical interaction between apoA-I and ABCA1 results in the formation of a phospholipid-apoA-I complex that promotes cholesterol efflux [121]. Three phospholipids, including CL, lysoCL 1, and 2 (LC_1_ and LC_2_), which together contribute only a small fraction of the total cellular phospholipid content, were found to be enriched up to fivefold in TD fibroblasts compared to wild type cells [122]. This finding suggests that phospholipid and cholesterol efflux may be coregulated and, therefore, dually impaired in TD cells. Additionally, it is possible that increased CL may play an as yet uncharacterized regulatory role in cholesterol trafficking and efflux.

## 4. CL Plays a Role in Cellular Events and Pathways That Are Important for Maintaining Cardiovascular Health

### 4.1. Mitochondrial Function

#### 4.1.1. Mitochondrial Dysfunction and CVD

To support the normal function of the heart, cardiomyocytes have a high mitochondrial density that comprises about 30% of the total intracellular volume [123]. This allows cardiomyocytes to produce ATP quickly to satisfy the high demand for energy. Even subtle alterations in mitochondrial function or membrane potential can cause a significant change in cardiomyocyte energy production and further harm cardiovascular health.

As discussed in Section 3.4, mitochondrial dysfunction and ROS play a causative role in the pathogenesis of myocardial ischemia-reperfusion injury. Mitochondrial dysfunction and related morphological abnormalities, ROS generation, and altered mitochondrial permeability transition pore and mitochondrial Ca^2+^ storage also contribute to the development of diabetic cardiomyopathy [124–126], dilated cardiomyopathy [127–129], dystrophic cardiomyopathy [130, 131], and hypertrophic cardiomyopathy [132–134]. Mitochondrial dysfunction is also linked to the development of HF, as demonstrated in the hamster [135]. The role of mitochondrial dysfunction as a cofactor accelerating the progression of existing CVD to HF has been addressed elsewhere [136, 137].

#### 4.1.2. CL Deficiency Leads to Mitochondrial Dysfunction

CL interacts with many inner mitochondrial membrane proteins, including electron transport chain (ETC) complex proteins that are components of complex I [62, 138], complex III [61, 138–140], complex IV [61, 139, 140], complex V [141], cytochrome c [142], and transporter proteins such as the ADP-ATP carrier [143], pyruvate carrier [144], and phosphate carrier [145]. Thus, CL deficiency can negatively impact the activity and efficiency of these proteins. Several studies demonstrate that ROS-induced CL oxidation causes concomitant inactivation of complexes I, III, and IV [146–148].* In vitro* studies indicate that adding CL liposomes, but not PE, PC, or oxidized CL liposomes, prevents ETC complex defects caused by CL oxidation [146]. In addition to interactions with single complexes, CL is required for the proper assembly and stability of ETC supercomplexes. In mammalian mitochondria, supercomplexes are comprised of complex I associated with complex III dimers and up to four monomers of complex IV [148]. Yeast mitochondria, which lack complex I, contain small supercomplexes of complex III dimers. Large supercomplexes are characterized by two small supercomplexes associated with complex IV [148]. CL is required for the assembly and stability of these supercomplexes. Supercomplexes of complexes III and IV are destabilized in yeast* crd1Δ* cells as detected by CN-PAGE [61, 140]. In lymphoblast cells of BTHS patients, complex IV readily dissociates from the supercomplex, and I/III supercomplex levels are decreased [149]. In addition to the impact of CL on the respiratory chain, CL deficiency also leads to other manifestations of mitochondrial dysfunction such as defective protein import and mitophagy, as discussed below.

#### 4.1.3. Mitochondrial Pharmaceutics in CVD

Because mitochondrial dysfunction plays a pivotal role in the pathogenesis and progress of CVD, the field of mitochondrial pharmaceutics is rapidly expanding [150]. Therapeutics that target heart mitochondria, including synthetic peptides (SS peptide family) [151–153], superoxide dismutase mimetics [154], and triphenylphosphonium- (TPP-) ligated antioxidants such as vitamin E [155], ubiquinone [156], and lipoic acid [157], exhibit promise in alleviating mitochondrial damage in CVD. Several of these drugs are currently being tested in clinical trials [150].

### 4.2. Mitochondrial Protein Import

More than 98% of mitochondrial proteins are encoded in the nucleus, synthesized in the cytosol as precursors, and imported into the mitochondria [158]. Thus, mitochondrial protein import is essential for maintaining normal mitochondrial function. As discussed above, a link between defective mitochondrial protein import and CVD was suggested by mutations in the DNAJC19 gene in DCMA syndrome. Two* in vitro* studies showed that the unfolding of an artificial mitochondrial protein precursor by CL was required for binding to isolated yeast mitochondrial outer membranes or liposomes. These findings were the first to demonstrate a mechanistic link between CL and protein import [105, 106]. A more direct demonstration of the role of CL in mitochondrial protein import was shown by decreased protein import in the yeast CL mutant* crd1Δ* [108]. CL was also shown to be involved in the biogenesis of mitochondria outer membrane protein import complexes [104]. Functional assays of precursor binding to the TOM complex, the translocase of the mitochondrial outer membrane, and the SAM complex, the outer membrane sorting and assembly machinery, revealed partially impaired precursor binding in CL mutants [104]. Loss of CL also leads to defective import of mitochondrial ATPase subunit precursors, which are located in the inner membrane or matrix [108].

### 4.3. Autophagy/Mitophagy

#### 4.3.1. Autophagy/Mitophagy as a Protective Mechanism against Cardiac Aging and Ischemia-Reperfusion

Autophagy refers to the cellular process in which cytoplasmic contents are delivered into the lysosome or vacuole for degradation. Autophagy is further classified as selective and nonselective autophagy [159]. Various types of selective autophagy have been identified, including mitophagy, pexophagy, lipophagy, nucleophagy, lysophagy, reticulophagy/ER-phagy, and ribophagy [160]. Mitophagy is the selective degradation of mitochondria by autophagy [161]. Mitophagy and autophagy are generally not distinguished in studies of CVD and will be discussed together here.

Numerous studies link autophagy to CVD. In the heart, autophagy is an important housekeeping process that is essential for maintaining cardiac health [162]. Deletion of* ATG5*, the gene encoding a protein that regulates phagophore expansion, is known to result in cardiomyopathy in mice [163]. Autophagic activity declines with age, and decreased or impaired autophagy leads to accumulation of proteins and damaged mitochondria, contributing to cardiac aging [164].

As early as the 1970s, autophagy was shown to be increased during ischemia [165]. After decades of research, the relationship between autophagy and cardiovascular physiology is only partially clear. As discussed above, damage to mitochondria is a hallmark of ischemia. During mild and chronic ischemia, mitophagy is increased as an adaptive and protective strategy to eliminate damaged mitochondria [166–168]. Increased autophagy is accompanied by decreased apoptosis during ischemia, suggesting that autophagy limits apoptotic necrosis of cardiomyocytes [166, 169]. Many studies implicate the involvement of AMPK activation in triggering autophagy/mitophagy during ischemia [170–172], although this is not conclusive [173]. Following reperfusion, autophagy is even more dramatically increased in animal models [166, 174, 175] and primary neonatal cardiomyocytes [169], having a detrimental effect that is at least partially mediated by activation of Beclin-1, the protein required for autophagosome formation [169, 170, 176].

#### 4.3.2. CL Is Needed for Maintaining Normal Mitophagy

CL is reported to externalize the outer mitochondrial membrane as an elimination signal for mitophagy in neuronal cells and to bind the microtubule-associated protein 1 light chain 3 (MAP1LC3/LC3), the marker protein of autophagic membranes. Binding induces recognition of mitochondria as the cargo by the autophagic machinery [177, 178]. The role of CL in mitophagy is supported by the finding that ALCAT1-catalyzed remodeling of CL with aberrant acyl groups leads to defective mitophagy in hepatocytes [179]. Interestingly, the autophagy-related protein Beclin-1 is preferentially enriched in lipid membranes that contain high concentrations of CL [180]. Deletion of* ATG5*, which is essential for autophagy, results in cardiomyopathy in mice [163]. These findings invite speculation that loss of CL and defective CL remodeling may contribute to the development of cardiomyopathy by a mechanism related to perturbation of mitophagy.

### 4.4. The PKC Pathway

#### 4.4.1. The Role of PKC in Cardiovascular Health

Protein kinase C (PKC) is a family of protein kinases that regulate the function of other proteins through specific phosphorylation of hydroxyl groups on threonine and serine residues. Human cells have fifteen PKC isozymes [181]. Overstimulation of PKC*α*, PKC*β*, PKC*δ*, or PKC*ε* results in hypertrophy of cardiomyocytes through activation of the extracellular signal-related kinase (ERK) pathway [182]. However, during ischemia preconditioning, PKC*α*, PKC*δ*, PKC*ε*, and PKC*η* have been shown to translocate to the active membrane pool and perform cardioprotective functions [182]. Activation of PKC*δ* results in intracellular pH changes and viability protection; activation of PKC*η* protects against myocardial stunning; activation of both PKC*δ* and PKC*η* provides global myocardial protection against necrosis, acidosis, and myocardial stunning [183]. Blocking the phosphatidylinositol-specific phospholipase C- (PI-PLC-) induced translocation of PKC*α*, PKC*ε*, and PKC*η* during ischemia impairs myocardial recovery [184]. Therefore, PKC isozymes have dual functions in the pathogenesis and progression of CVD. However, unlike other PKC isozymes that have dual roles in different CVDs, PKC*η* is mainly reported to play a cardioprotective role during ischemia.

#### 4.4.2. Loss of CL Leads to Defective PKC

During hyperthermia-induced apoptosis, PKC*δ* phosphorylates phospholipid scramblase 3 (PLS3), which then induces CL translocation from the inner to outer mitochondrial membrane [185–187]. This series of reactions is considered an indicator of both apoptosis and autophagy. The relationship between CL and PKC appears to be interdependent. While CL translocation is regulated by PKC*δ*, CL may also be a regulator of the PKC pathway. Studies in yeast, which have only one PKC (Pkc1), show that loss of CL may lead to defects in the activation of the PKC pathway [188]. Human PKC*η* is the only human PKC isozyme that can complement the defects caused by deletion of* PKC1* in yeast through activation of the same protein kinase cascade [189]. This suggests that PKC*η* shares both functional homology and structural homology with Pkc1. Extrapolating from the finding in yeast that CL plays a role in PKC pathway activation, the cardioprotective role of PKC*η* activation during ischemia preconditioning may be dependent on CL.

## 5. Conclusion

As discussed above, CL plays important roles in cellular processes and pathways that are crucial for heart function, including mitochondrial function, mitochondrial protein import, autophagy/mitophagy, and the PKC pathway. CL synthesis and remodeling are highly regulated under physiological conditions, and perturbation of this regulation results in aberrant CL profiles in associated cardiac disorders, including cardiomyopathy, myocardial ischemia-reperfusion injury, HF, atherosclerosis, and Tangier disease. However, the mechanisms linking CL to these pathologies remain to be elucidated.

Mechanisms underlying the role of ox-CL in the pathogenesis of myocardial ischemia-reperfusion injury and atherosclerosis have been suggested. Apoptosis and necrosis during ischemia-reperfusion may result from decreased binding of cytochrome c to ox-CL [84], which likely leads to the release of cytochrome c and to increased permeability of the mitochondrial membrane to apoptosis factors [82, 190]. In addition, ox-CL also functions as an antigen to stimulate proinflammatory effects during the formation of atherosclerosis.

The relative contribution of CL/MLCL levels and acyl composition in maintaining respiratory chain function and cardiovascular health is not understood. Many studies have suggested that the lack of unsaturated L4-CL may be the cause of the pathology in BTHS [43, 44]. Consistent with the importance of CL acyl composition, knockdown of* DNAJC19* alters the acyl chain composition of CL without influencing the total CL level [109]. However, the finding that growth and respiratory defects of the yeast* taz1* mutant are rescued by deletion of* CLD1*, which restores CL/MLCL levels without generating remodeled CL, suggests that CL/MLCL levels are more important for mitochondrial function than CL acyl composition [53, 54].

In summary, elucidating the mechanisms whereby CL regulates cardiac function remains a vastly unexplored and exciting frontier that holds the promise of potential new therapies to treat cardiac disorders.
